# Challenges in Tracking of Fluorochrome-Labelled Nanoparticles in Mice via Whole Body NIRF Imaging

**DOI:** 10.3390/nano10030596

**Published:** 2020-03-24

**Authors:** Florian Gaffron, Andrea Tilch, Cordula Grüttner, Anja Kowalski, Martin Kramer, Ulf Teichgräber, Ingrid Hilger

**Affiliations:** 1 Institute for Diagnostic and Interventional Radiology, Jena University Hospital—Friedrich Schiller University Jena, D-07740 Jena, Germany; florian@gaffron.org (F.G.); andreatilch@posteo.de (A.T.); ulf.teichgraeber@med.uni-jena.de (U.T.); 2 Micromod Partikeltechnologie GmbH, Friedrich-Barnewitz-Str. 4, D-18119 Rostock, Germanykovalski@micromod.de (A.K.); 3 Department of Veterinary Clinical Sciences, Small Animal Clinic, Justus- Liebig-University, D-35390 Gießen, Germany; martin.kramer@vetmed.uni-giessen.de

**Keywords:** fluorescence, nanoparticles, iron oxide, nanoparticle tracking, nanoparticle labelling

## Abstract

Fluorochrome-labelled iron oxide magnetic nanoparticles (MNP) have been of great help in elucidating biological processes. Here, we used dually-fluorochrome-labelled MNP and studied to what extent fluorescence detection could reflect their fate in living animals. One day after application in mice (200 µmol Fe/kg body weight), the fluorescence of the dye attached to the core (DY-730) was very prominent and in agreement with the increase of iron in the liver and spleen of mice, but inconspicuous at time points thereafter. We attribute this fluorescence behavior to early degradation processes of the MNP´s core in the cellular lysosomal compartment. In contrast, the fluorescence of the dye DY-555 stuck to the PEG coating was not detectable in vivo. In summary, labelling of MNP with dyes at their metallic core could be of help when detecting first incidences of MNP biodegradation in vivo, as opposed to dyes attached to the MNP coating.

## 1. Introduction

Numerous suggestions have been made for the utilization of iron oxide magnetic nanoparticles (MNP) for biomedical applications, such as for magnetic targeting [1], magnetic hyperthermia [2], magnetic particle imaging [3], and magnetic resonance imaging [4] purposes, just to mention a few examples. From the chemical point of view, iron oxide MNP are based on maghemite, magnetite, and nowadays additionally of mixed ferrites [5]. To assure a superparamagnetic behavior of the many applications, the core is usually coated with polymers. Among them, poly-ethylene glycol (PEG) coating has gained particular attraction, since PEG is a hydrophilic molecule (repeating ethylene ether units), which reduces charge-based MNP interactions with proteins compared to the uncoated counterparts; it further reduces uptake by the mononuclear phagocyte system (MPS) and increases circulation time (e.g., [6]). In numerous studies on MNP therapeutic efficacy, the biodistribution, degradation, and off-target effects have been assessed as crucial parameters for further decisions for the onset of further studies towards clinical translation. 

In several preclinical studies, the fluorescently-labelled MNP have been used not only to understand the localization in target cells, but also to assess their biodistribution in vivo [7]. For these purposes, dyes and iron oxide cores have been encapsulated into a polymer matrix; alternatively, the dye has been attached to the MNP core or at its surface coating [8]. Such studies have been conducted with the assumption that the fluorescence label accurately reflects the MNP localization in the body and that the fluorescence intensity in the region of interest actually correlates with the amount of MNP.

Nanoparticle processing in vivo includes biotransformation, degradation, bioassimilation, and elimination [9]. In particular, it is known that macrophages (Kupffer cells) of the liver and spleen [10] play a crucial role in iron oxide MNP degradation. Specifically, after endocytotic uptake, the MNP are accumulated in lysosomes, where its degradation occurs by the loss of the protective layer on the core surface, by chemical etching of the crystal, by release of free Fe^3+^ ions, and finally by the formation of iron–protein complexes [11]. Iron taken up by macrophages easily enters the iron recycling pathways for hemoglobin and ferroprotein synthesis, as well as for iron storage [12,13]. 

The fate of fluorochrome dyes is less well understood. In relation to cyanine and hemi-cyanine dyes with good in vivo imaging properties, it is rather known that they can be subjected to opsonization processes when administered intravenously and that they are excreted via the liver, bile, and feces and/or the kidney depending on their physicochemical structures [14].

The aforementioned features raise the question as to what extent fluorescence tracking reflects the MNP fate in living animals. For this reason, we studied the fate of iron core of MNP and two different fluorochrome labels in terms of organ distribution in dependence on time after systemic application in mice. To further elucidate the impact of lysosomal environment on MNP degradation, several in vitro studies were performed. For these purposes, we used a polyethylene (PEG)-coated iron oxide MNP formulation which was strongly labelled with one hemi-cyanine dye emitting at 700-nm wavelength range at the MNP´s core, and another one emitting at the 500 nm wavelength range coupled to the hydrophilic PEG shell of the MNP coating. 

## 2. Materials and Methods 

MNP synthesis. BNF-Dextran core particles with NH_2_ groups on the surface (micromod Partikeltechnologie GmbH, Germany) (84-01-102, 10 mL, 10 mg/mL) were mixed with 1 mL 10× PBS buffer (0.1 M, pH = 7.4) and 123 µL DY-730-NHS ester solution (2 mg/mL in DMSO). DY-730-NHS ester (Dyomics GmbH, Jena, Germany) has an absorption of 734 nm and emission of 755 nm. The MNP suspension was shaken for 2 h at ambient temperature and washed three times with each 10 mL PBS buffer (0.01 M, pH =7.4) using a magnetic separator (LifeSep® 15SX, Sigma Aldrich, Munich, Germany). An amount of 6 mg EDC and 157 mg of HOOC–PEG–COOH (IRIS Biotech GmbH, Marktredwitz, Germany) (MW: 5 kDa) were dissolved in each 2 mL PBS buffer (0.01 M, pH = 7.4), mixed, and incubated for 10 min at 50 °C. The activated PEG derivative was transferred to the NMP suspension, shaken for 2 h at ambient temperature, and finally washed three times with each 10 mL PBS buffer (0.01 M, pH = 7.4) using a magnetic separator. The terminal PEG–COOH groups were activated by addition of 6 mg EDC (dissolved in 1 mL PBS buffer; 45 min at ambient temperature) and 10 µL tris(2-aminoethyl)amine. After 2 h of shaking at ambient temperature, the MNP suspension was washed three times with PBS (10 mL each, 0.01 M, pH = 7.4) using a magnetic separator. The NH_2_ groups on the particle shell were reacted with 120 µL DY-555-NHS ester solution (2 mg/mL in DMSO). DY-555-NHS ester (Dyomics GmbH, Jena, Germany) has an adsorption of 547 nm and an emission of 573 nm. Finally, the MNP suspension was washed multiple times with PBS (10 mL each, 0.01 M, pH = 7.4) using a magnetic separator until the supernatant showed no absorption at 547 nm. The particle suspension was filtered through a 0.2 µm polyethersulfone syringe filter. The hydrodynamic particle diameter was shown to be of 144 nm (polydispersity index: 0.1) as measured by dynamic light scattering (Zetasizer Nano-ZS90, Malvern Instr., Ltd., Malvern, UK), the magnetization was of 49 Am^2^/kg iron (80 kA/m); saturation magnetization, >76 Am^2^/kg iron (>800 kA/m); coercive field, 0.45 kA/m; iron oxide core was composed of single magnetite crystals with a diameter of 15 nm, and they were packed together to form a core of 80 to 100 nm. 

Animal studies. The experiments were carried out in accordance with international guidelines on the ethical use of animals and they were approved by the regional animal care committee (Thüringer Landesamt für Verbraucherschutz, Bad Langensalza, Germany). Female athymic nude mice (Envigo RMS) were humanely cared for during the whole experimentation period. They were maintained under artificial day–night cycles (14/20 h light–dark cycles; 21 ± 2 °C room temperature, 55 ± 5% environment humidity) and received food and water ad libitum. Animals were randomly distributed into 2 groups: Animals of group 1 (*n* = 5) received dually-labelled MNP-D(DY-730)-PEG-5kDa(DY-555) (200 µmol Fe/KG body weight, sequential injections) via the tail vein. Noninjected animals of group 2 (n = 5) were used as controls for organ fluorescence (DY-730 and DY-555) and iron content. At the defined time points (1 day (24 h), and 2 to 6 months after MNP-injection), animals were killed, dissected, and semiquantitative fluorescence intensity (region of interest (ROI): 916 pixels) of the mentioned dyes in the excised liver and spleen was determined using the MaestroTM In-Vivo Imaging System (Cambridge Research and Instrumentation Inc. (Cri), 01801 Woburn, MA, USA). Detection of DY-750 and DY-555 fluorescence in tissues was done via utilization of the Maestro Excitation/Emission Filter Deep Red (exc. 671–705 nm, em. 750 nm long pass) and the Maestro Excitation/Emission Green Filter (exc. 503–555 nm, em. 580 nm) set, respectively. The acquired composite spectral fluorescence data were unmixed into the spectrum of the respective fluorochromes (for details see [15]). Moreover, organ tissue (liver and spleen) was embedded into paraffin and processed for histochemical analysis (Prussian blue staining). 

To assess the impact of the lysosomal environment on our dually fluorochrome-labelled MNP on their spectroscopic features, MNP were incubated at 37 °C in artificial lysosomal fluid (ALF), which was composed of citrate buffer pH 4.5 and diverse endo-lysosomal metabolic products according to a previous publication [16]. To control the citrate iron chelating effects and the absence of lysosomal metabolites, citrate buffer at pH 4.7 without supplements was used. To control the effect of low pH and the presence of citrate, ddH_2_O was used as suspension medium (pH 7). 

To estimate the impact of free Fe^3+^ resulting from degradation of the MNP core in the lysosomal compartment on the fluorescence of DY-730 and DY-555, those dyes were solubilized in ALF, citrate buffer (CB), or ddH_2_O (15 nmol in 2 mL) in absence or presence of excess of Fe^3+^ (1 mmol). ROI-based (3346 pixels) semiquantitative determination of fluorescence intensities was performed as described above.

## 3. Results

**Figure 1:**
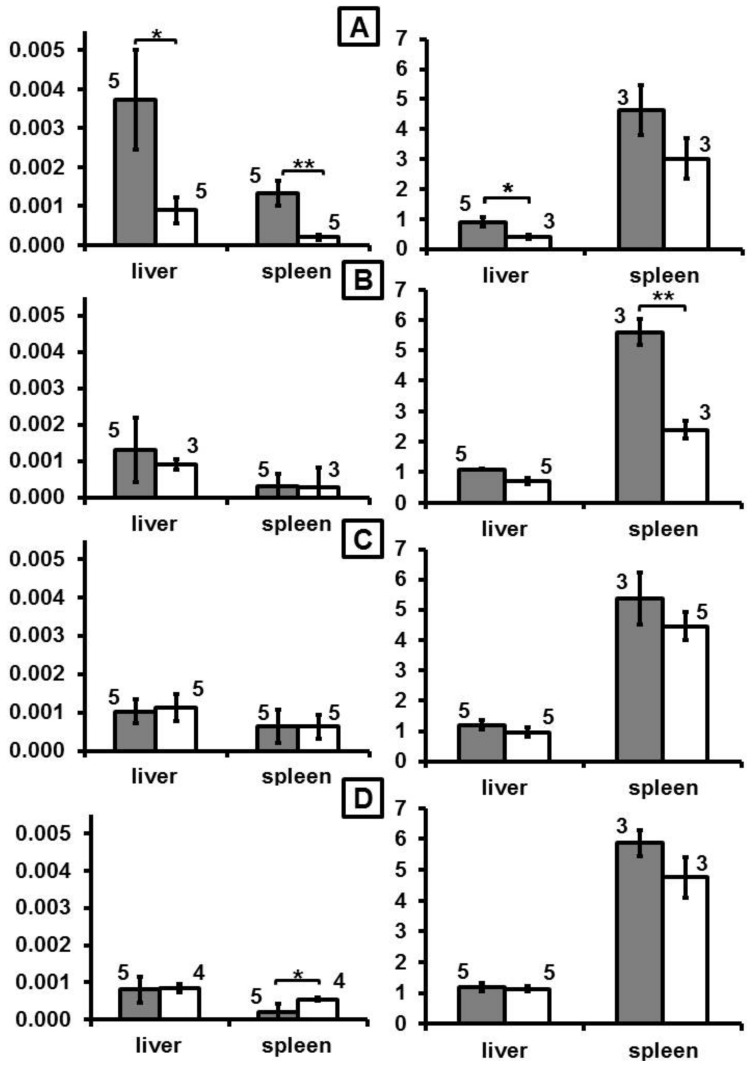
The time-dependent organ distribution fluorescence of the dye DY-730 attached to the iron oxide magnetic nanoparticles and the iron concentration in the organs. (**A**–**D**): 0, 2, 4, and 6 months after MNP injection in mice, respectively. Left panel: semiquantitative fluorescence intensity (em: 755 nm, arbitrary units per s, n = 5); right panel: iron content (mg iron per g tissue, n = 3). Nude mice were injected with dually-labelled MNP-D(DY-730)-PEG-5kDa(DY-555) (200 µmol Fe/kg body weight). Gray and white bars: after MNP injection or native controls, respectively. At the defined time points, animals were killed and semiquantitative fluorescence intensities of each fluorochrome in excised organs were determined. ROI: 916 pixels n = 5, mean and standard deviation of the mean, * p < 0.05; ** p < 0.01, Tukey post-hoc Test. PEG: poly-ethylene glycol.

**Figure 2:**
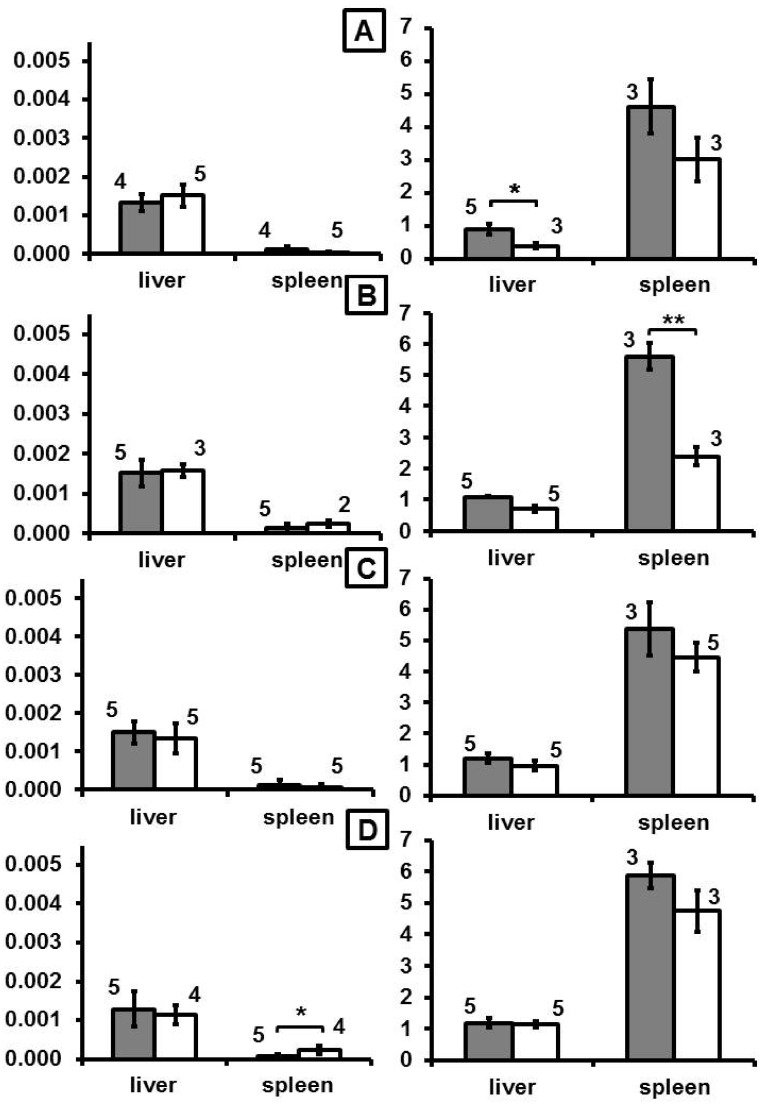
The time-dependent organ distribution fluorescence of the dye DY-555 attached to the MNP and the iron concentration in the organs. (**A**)–(**D**): 0, 2, 4, and 6 months after MNP injection in mice, respectively. Left panel: semiquantitative fluorescence intensity (em: 573 nm, arbitrary units per s, n = 5); right panel: iron content (mg iron per g tissue, n = 3). Gray and white bars: after MNP injection or native controls, respectively. Nude mice were injected with dually-labelled MNP-D(DY-730)-PEG-5kDa(DY-555) (200 µmol Fe/kg body weight). At the defined time points, animals were killed and semiquantitative fluorescence intensities of each fluorochrome in excised organs was determined. ROI: 916 pixels, n = 5, mean and standard deviation of the mean, * p < 0.05; ** p < 0.01; Tukey post-hoc Test.

**Figure 3:**
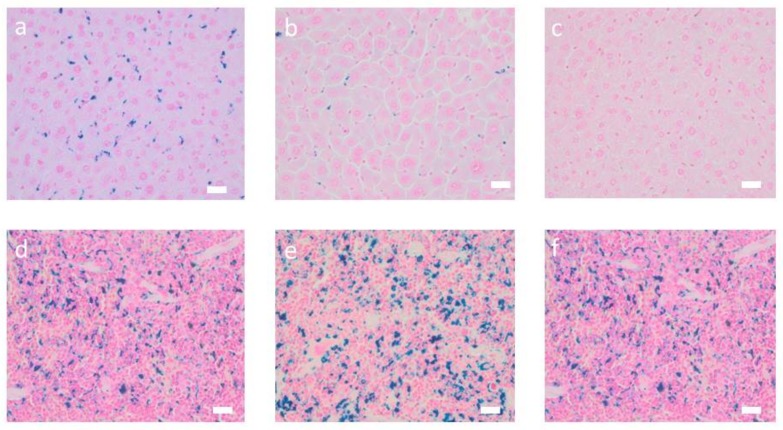
Animals injected with MNP-D(DY-730)-PEG-5kDa(DY-555) show distinct iron depositions in liver and spleen. Iron oxide deposits in Kupffer cells of the liver and hepatocytes (**a**–**c**) and spleen (**d**–**f**) of mice injected with MNP-D(DY-730)-PEG-5kDa(DY-555), 200 µmol Fe/KG body weight. (**a**,**d**): 0 months; (**b**,**e**): 2 months after MNP injection; (**c**,**f**): non-MNP injected animals. Prussian blue staining of liver and spleen tissue. Bars: 20 µm.

After intravenous application, mice showed a distinct increase of DY-730 fluorescence mainly in the liver (almost 3-fold of control, p < 0.05) and in the spleen (more than 3-fold of control, p < 0.01) at one day (month 0 in Figure 1, and Supplementary Figure S1) after MNP application. Later on (at month 2 and later), fluorescence increase emerging from dye DY-730 was inconspicuous (no statistical difference, Figure 1). In comparison to that, iron content increased 1-fold (p < 0.05) and 0.5-fold (compared to nontreated controls) in the liver and the spleen at month 0. Later on, at month 2, increase of iron was only low in the liver (0.3-fold), but very prominent in the spleen (1.5-fold of control). At later periods of time (i.e., months 4 and 6), the iron levels were not distinctly different from controls anymore (Figure 1). With regard to the fluorescence of the fluorochrome dye DY-555, almost no distinct fluorescence was seen at any observation period (Figure 2). Prussian blue staining of liver and spleen showed that iron was prominent in hepatocytes surrounding liver veins (sinuses) as well as in Kupffer cells of liver and spleen (Figure 3).

**Figure 4:**
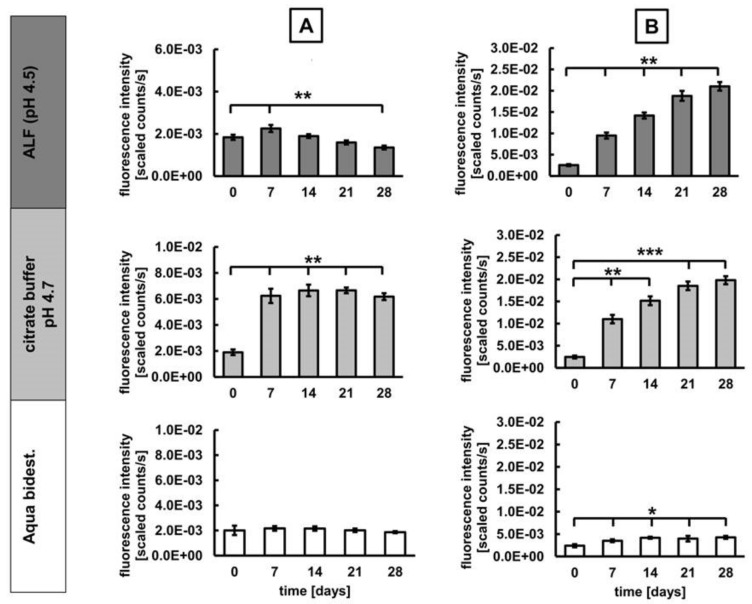
Fluorescence of DY-730 and DY-555 of the dually-labelled MNP-D(DY-730)-PEG-5kDa(DY-555) behave differently in acidic conditions resembling lysosomes. (**A**) Fluorescence emission (755 nm) of DY-730 attached to the MNP core, (**B**) Fluorescence emission (573 nm) of DY-555 attached to the PEG-coating of the nanoparticles. MNP were suspended in 1% agarose in ddH_2_O and incubated at 37 °C und 5% CO_2_ for the period of time indicated, semiquantitative fluorescence analysis of ROIs (3346 pixels each), mean and standard deviation of the mean of n = 3, * p < 0.05; ** p < 0.01; *** p < 0.001.

With regard to the NMP´s spectroscopic behavior in different biological media, we observed the following: In ALF medium, a transient increase of DY-730 fluorescence was seen at days of incubation (p < 0.05), whereas the increase of DY-555 fluorescence was much more prominent (p < 0.01). The citrate buffer control showed a prominent dye DY-730 fluorescence increase, which indicates that the presence of endo-lysosomal metabolites attenuated dye hydrolysis. Moreover, citrate iron chelation in the acidic environment (high proton concentration) favored fluorescence emission of DY-730, but had almost no effect on the DY-555 fluorescence. In water (low pH control), DY-730 fluorescence remained unchanged, but fluorescence of DY-555 increased (p < 0.05) with increasing time of incubation (Figure 4).

**Figure 5:**
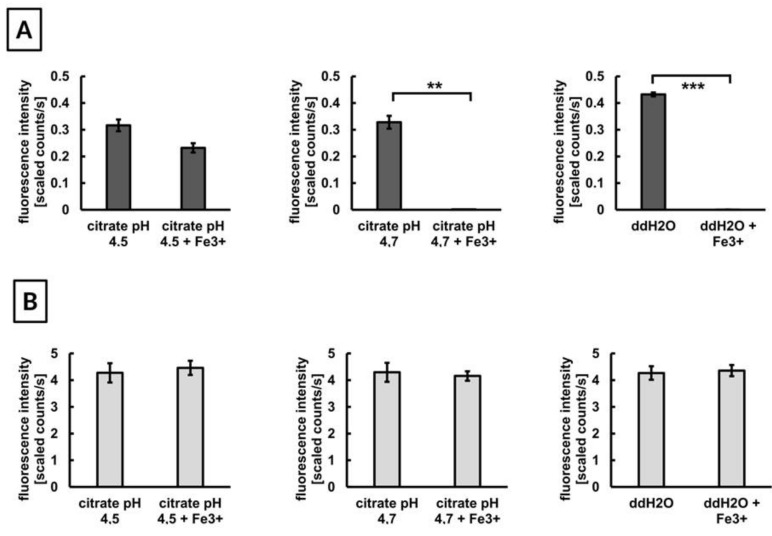
The fluorescence emission (755 nm) of DY-730 (**A**) is susceptible the presence of free Fe-ions whereas that of DY-555 (573 nm) (**B**) is not. Semiquantitative analysis of fluorescence intensity of the dyes after suspension in artificial lysosomal fluid—left, CB (citrate buffer)—center, or ddH_2_O (control)—right. 15 nmol dye in 2 mL suspension medium, in presence or not of 1 mmol Fe^3+^ and measurement of fluorescence intensity (ROI: 3346 pixel) immediately thereafter, mean and standard deviation of the mean, ** p < 0.01; *** p < 0.001.

The analysis of the fluorescence behavior of MNP in presence of excess free Fe^3+^—as it occurs during the iron oxide core corrosion process in the lysosomal compartment—showed that fluorescence of DY-730 slightly decreased in ALF, whereas in citrate buffer it was rather abolished. This indicates that the presence of excess Fe^3+^ in absence of endosomal metabolites is unfavorable for DY-730 fluorescence. This strong DY-730 fluorescence decrease was also seen in the presence of water, a finding which corroborates the notion that the DY-730 fluorescence quenching mainly emerges from the presence of free Fe^3+^. In contrast, fluorescence of DY-555 (36/G) was unaffected by the presence of free Fe^3+^, independent of the surrounding medium (water, ALF) (Figure 5).

## 4. Discussion

Our results basically show that our synthesis method was able to produce some MNPs with fluorochrome dyes strongly bound to core and other ones with high hydrolytic features to the PEG coating. The fluorescence of the core-bound dye (DY-730) in the liver and spleen of mice was in agreement with the increase of iron only at the early time point of examination. Based on the facts that i) MNP are opsonized and recognized by MPS [17], ii) that they are mainly taken up by Kupffer cells of the liver and spleen (our data and from other groups [10]), and iii) that the MNP degradation takes place in their lysosomal environment, our examinations (in vitro) show that there was a transient fluorescence increase of the core-bound dye DY-730 in the lysosomal environment. Moreover, citrate-iron chelating seems to favor core dye fluorescence, whereas excess of free Fe^3+^ is controversial to it. Opposed to dye DY-730, the fluorescence of the dye DY-555 stuck to PEG coating was not detectable in mice, but it showed a distinct increase in the lysosomal environment and no sensitivity in the presence of excess free iron in vitro.

With consideration of the fluorescence of the dye attached to the MNP core, which increased in the liver and spleen at 24 h (month 0) after application, we postulate that it reflects first incidences of MNP biodegradation in lysosomes. Namely, this fluorescence increase seems to be mainly the result of fluorescence dequenching in the acidic environment. Potential reasons are as follows: a) As a consequence of a more or less intense degradation of PEG in lysosomes, e.g., favored by the presence of reactive oxygen species (ROS) [18], the iron oxide core is susceptible of corrosion [19] and consequently the dye DY-730 to hydrolysis. b) It has been shown that degradation of iron oxide cores in acidic medium takes approximately 25 to 30 days [20] and, in our study, the time point between the last sequential iron oxide application and the first measurement is 1 day. c) Our own examinations show that upon exposure of our dually-labelled MNP to a lysosomal environment, DY-730 fluorescence is initially dequenched (e.g., 7 days incubation, in vitro). In vivo, DY-730 fluorescence decreased at later observation periods (month 2 and later on), presumably due to the presence of excess free Fe^3+^ in the lysosomal compartment (the relative proportion of iron per MNP is much higher than that of a fluorochrome dye) and/or degradation processes of the hemi-cyanine dye. Additionally, the presence of lysosomal metabolites seems to further contribute to the fluorescence quenching of DY-730, according to our in vitro results. 

The different behavior of organ DY-730 fluorescence of the MNP´s core and iron content noted in the in vivo situation indicate that both entities undergo separate degradation mechanisms with the onset of MNP degradation. With respect to the (hemi)cyanine dyes: Despite several investigations performed with intravenous application, the degradation of (hemi)cyanines in endo-lysosomes are unclear. Hereto, the involvement of CYP3A4 is conceivable [21], since macrophages are able to express CYP3A4 and other CY450 proteins [22]. Moreover, as macrophages and hepatocytes express membrane influx/efflux proteins (e.g., P-gp [23]), hemi-cyanines or their lipophilic metabolites might well be exported from macrophages to hepatocytes (e.g., via membrane efflux proteins) for further biliary clearance [14]. With consideration of the fate of the MNP´s iron: a massive biodegradation of the iron oxide nanoparticles at endosome sites has been determined by the loss of nanospecific magnetic properties in former studies (e.g., within 10 days, stem cell spheroids [24], and the intra-lysosomally released iron was shown to be transported out of the endo-lysosomal compartment, e.g., via endosomal transmembrane transport (DMT1), for incorporation into metalloproteins, ferritin (detoxification), and/or transport out of the cell via ferroportin-1 (FPN1 [25]). Interestingly, ferritin is capable of caging Fe^3+^ under acidic conditions [20], and ferritin-loaded Kupffer cells might favor MNP dye DY-730 fluorescence. On the other hand, neosynthesis of iron oxide nanoparticles from nanodegradation products within the endosomes has been postulated and, interestingly, H-subunits of ferritin may be involved in this process [26]. Under these conditions, one would expect an improved fluorescence-based MNP localization in the liver and spleen. Taking this line of arguments together, tracking of MNP in vivo via DY-730 fluorescence of the dye core is only feasible in early steps of degradation in cells of the MPS. 

In the case of the DY-555 stuck to the PEG coating with high hydrolytic susceptibility, no dye fluorescence was seen in organs of the MPS in liver and spleen. There are some possible explanations for this finding. The first explanation is that DY-555 dyes could have been detached from the MNP already in the blood and/or they have already been injected as free molecules (at least in parts) at a time point before the first measurement started. There are several supporting observations for this argumentation: a) Dyes attached to the PEG coating are, per se, more susceptible to hydrolysis than dyes placed at the MNP´s core. b) Dye DY-555 is a rhodamine molecule with a spirocyclic structure, which hydrolyses more readily than DY-730. c) DY-555 carries a negative sulphonic group, and a continuous change of negative zeta-potential of MNP was detected already in water, which can be interpreted as an additional indication for dye hydrolysis. d) The second explanation is that the fluorescence emission of DY-555 (em.: 573 nm) might still be too narrow to that of other liver and spleen tissue intrinsic fluorochromes in order to be adequately discerned via spectral unmixing procedures (near infrared optical window; e.g., [27]). This means that in the in vivo situation in mice, we could not take benefit of the favorable spectroscopic behavior of DY-555 coupled to our dually-labelled MNP, which has been detected in the lysosomal environment in vitro. 

We observed that the iron content increased prominently in the spleen at month 2 (compared to month 1) after MNP-application. This is an indication that iron is being redistributed when the MNP´s core dyes (DY-730) are not visible with imaging methods anymore, presumably because of their degradation. Since at the same time the iron content decreased in the liver (compared to month 1 after MNP application), iron redistribution mechanisms between the liver and the spleen are conceivable. There is little known about such redistribution procedures, but the following factors could have contributed to it: i) Iron is exported out of the Kupffer cells via the iron exporter ferroportin (Fpn) and taken up by macrophages of the red pulp in the spleen. ii) Macrophages of red pulp of the spleen are known to be highly specialized in iron recycling (CD163, ferroportin, heme oxygenase-1). iii) Macrophages of the red pulp could be involved by a yet unknown mechanism in the immune-mediated clearance of senescent Kupffer cells, which were carrying ferritin [28]. 

Taken together, we were able to show that tracking of iron oxide MNP with fluorescent dyes is possible in laboratory animals (e.g., via in vivo imaging methods), when a hemi-cyanine dye (DY-730) is attached to the MNP´s core. Hereto, the presence of fluorescence signals in the liver could be an indication for early degradation processes in lysosomes of macrophages in organs of the MPS. Instead, the coupling of highly hydrolytic dyes to the PEG coating might be unfavorable for MNP tracking purposes in vivo, even though they apparently demonstrate appropriate spectroscopic features in the lysosomal environment.

## Supplementary Materials

The following are available online at https://www.mdpi.com/2079-4991/10/3/596/s1, Figure S1: Macroscopic light and superimposed color-coded fluorescence images of liver and spleen after injection of dually-labelled MNP-D(DY-730)-PEG-5kDa(DY-555).

## References

[1] Alexiou C., Arnold W., Klein R.J., Parak F.G., Hulin P., Bergemann C., Erhardt W., Wagenpfeil S., Lübbe A.S. (2000). Locoregional cancer treatment with magnetic drug targeting. Cancer Res..

[2] Sanhaji M., Göring J., Couleaud P., Aires A., Cortajarena A.L., Courty J., Prina-Mello A., Stapf M., Ludwig R., Volkov Y. (2019). The phenotype of target pancreatic cancer cells influences cell death by magnetic hyperthermia with nanoparticles carrying gemicitabine and the pseudo-peptide NucAnt. Nanomed. Nanotechnol. Boil. Med..

[3] Khandhar A., Ferguson R.M., Arami H., Krishnan K.M. (2013). Monodisperse magnetite nanoparticle tracers for in vivo magnetic particle imaging. Biomaterials.

[4] Jin R., Lin B., Li D., Ai H. (2014). Superparamagnetic iron oxide nanoparticles for MR imaging and therapy: design considerations and clinical applications. Curr. Opin. Pharmacol..

[5] Wu L., Wang Y., James T.D., Jia N., Huang C. (2018). A hemicyanine based ratiometric fluorescence probe for mapping lysosomal pH during heat stroke in living cells. Chem. Commun..

[6] Van Vlerken L.E., Vyas T.K., Amiji M. (2007). Poly(ethylene glycol)-modified Nanocarriers for Tumor-targeted and Intracellular Delivery. Pharm. Res..

[7] Corem-Salkmon E., Margel S., Perlstein B. (2012). Design of near-infrared fluorescent bioactive conjugated functional iron oxide nanoparticles for optical detection of colon cancer. Int. J. Nanomed..

[8] García R.S., Stafford S., Gun’Ko Y.K. (2018). Recent Progress in Synthesis and Functionalization of Multimodal Fluorescent-Magnetic Nanoparticles for Biological Applications. Appl. Sci..

[9] Kolosnjaj-Tabi J., Lartigue L., Javed Y., Luciani N., Pellegrino T., Wilhelm C., Alloyeau D., Gazeau F. (2016). Biotransformations of magnetic nanoparticles in the body. Nano Today.

[10] Okon E., Pouliquen D., Okon P., Kovaleva Z.V., Stepanova T.P., Lavit S.G., Kudryavtsev B.N., Jallet P. (1994). Biodegradation of magnetite dextran nanoparticles in the rat. A histologic and biophysical study. Lab. Investig..

[11] Levy M., Lagarde F., Maraloiu V.-A., Blanchin M.-G., Gendron F., Wilhelm C., Gazeau F. (2010). Degradability of superparamagnetic nanoparticles in a model of intracellular environment: follow-up of magnetic, structural and chemical properties. Nanotechnology.

[12] Gammella E., Buratti P., Cairo G., Recalcati S. (2017). The transferrin receptor: the cellular iron gate. Metallomics.

[13] Ganz T. (2012). Macrophages and systemic iron homeostasis. J. Innate Immun..

[14] Hamann F.M., Brehm R., Pauli J., Grabolle M., Frank W., Kaiser W.A., Fischer D., Resch-Genger U., Hilger I. (2011). Controlled Modulation of Serum Protein Binding and Biodistribution of Asymmetric Cyanine Dyes by Variation of the Number of Sulfonate Groups. Mol. Imaging.

[15] Mansfield J.R., Hoyt C., Levenson R.M. (2008). Visualization of Microscopy-Based Spectral Imaging Data from Multi-Label Tissue Sections. Curr. Protoc. Mol. Boil..

[16] Stopford W., Turner J., Cappellini D., Brock T. (2003). Bioaccessibility testing of cobalt compounds. J. Environ. Monit..

[17] Hannon G., Lysaght J., Liptrott N.J., Prina-Mello A. (2019). Immunotoxicity Considerations for Next Generation Cancer Nanomedicines. Adv. Sci..

[18] Ulbricht J., Jordan R., Luxenhofer R. (2014). On the biodegradability of polyethylene glycol, polypeptoids and poly(2-oxazoline)s. Biomaterials.

[19] Wang C., Zhao T., Li Y., Huang G., White M.A., Gao J. (2016). Investigation of endosome and lysosome biology by ultra pH-sensitive nanoprobes. Adv. Drug Deliv. Rev..

[20] Volatron J., Carn F., Javed Y., Vuong Q.L., Gossuin Y., Luciani N., Charron G., Alloyeau D., Kolosnjaj-Tabi J., Gazeau F. (2016). Ferritin Protein Regulates the Degradation of Iron Oxide Nanoparticles. Small.

[21] Zhou W., Hu X.H., Tam K.Y. (2017). Systemic clearance and brain distribution of carbazole-based cyanine compounds as Alzheimer’s disease drug candidates. Sci. Rep..

[22] Anttila S., Hukkanen J., Hakkola J., Stjernvall T., Beaune P., Edwards R.J., Boobis A., Pelkonen O., Raunio H. (1997). Expression and localization of CYP3A4 and CYP3A5 in human lung. Am. J. Respir. Cell Mol. Boil..

[23] Cory T.J., He H., Winchester L.C., Kumar S., Fletcher C.V. (2016). Alterations in P-Glycoprotein Expression and Function Between Macrophage Subsets. Pharm. Res..

[24] Mazuel F., Espinosa A., Luciani N., Reffay M., Le Borgne R., Motte L., Desboeufs K., Michel A., Pellegrino T., Lalatonne Y. (2016). Massive Intracellular Biodegradation of Iron Oxide Nanoparticles Evidenced Magnetically at Single-Endosome and Tissue Levels. ACS Nano.

[25] Lane D., Merlot A., Huang M.L.-H., Bae D.-H., Jansson P., Sahni S., Kalinowski D., Richardson D.R. (2015). Cellular iron uptake, trafficking and metabolism: Key molecules and mechanisms and their roles in disease. Biochim. Et Biophys. Acta-Mol. Cell Res..

[26] Van De Walle A.B., Sangnier A.P., Abou-Hassan A., Curcio A., Hémadi M., Menguy N., Lalatonne Y., Luciani N., Wilhelm C. (2019). Biosynthesis of magnetic nanoparticles from nano-degradation products revealed in human stem cells. Proc. Natl. Acad. Sci. USA.

[27] Smith A.M., Mancini M.C., Nie S.M. (2009). BIOIMAGING Second window for in vivo imaging. Nat. Nanotechnol..

[28] Tchkonia T., Zhu Y., Van Deursen J., Campisi J., Kirkland J.L. (2013). Cellular senescence and the senescent secretory phenotype: therapeutic opportunities. J. Clin. Investig..

