# Supplementary material for: Challenges in Tracking of Fluorochrome-Labelled Nanoparticles in Mice via Whole Body NIRF Imaging
Source: Nanomaterials (Basel). 2020 Mar 24;10(3):596. doi: 10.3390/nano10030596 (PMC7153379; doi:10.3390/nano10030596)
Supplement: Supplementary file 1 [file nanomaterials-10-00596-s001.pdf]

Supplementary

Challenges in Tracking of Fluorochrome-Labelled Nanoparticles in Mice via Whole Body NIRF Imaging

Florian Gaffron [1], Andrea Tilch [1], Cordula Grüttner [2], Anja Kowalski [2], Martin Kramer [3], Ulf Teichgräber [1], and Ingrid Hilger [1,*]

[1] Institute for Diagnostic and Interventional Radiology, Jena University Hospital—Friedrich Schiller University Jena, D-07740 Jena, Germany; florian@gaffron.org (F.G.); andreatilch@posteo.de (A.T.); ulf.teichgraeber@med.uni-jena.de (U.T.)

[2] Micromod Partikeltechnologie GmbH, Friedrich-Barnewitz-Str. 4, D-18119 Rostock, Germany; gruettner@miromod.de (C.G.); kovalski@micromod.de (A.K.)

[3] Department of Veterinary Clinical Sciences, Small Animal Clinic, Justus- Liebig-University, D-35390 Gießen, Germany, martin.kramer@vetmed.uni-giessen.de

* Correspondence: ingrid.hilger@med.uni-jena.de; Tel.: +0049-3641-932-5921

Received: 28 January 2020; Accepted: 20 March 2020; Published: date

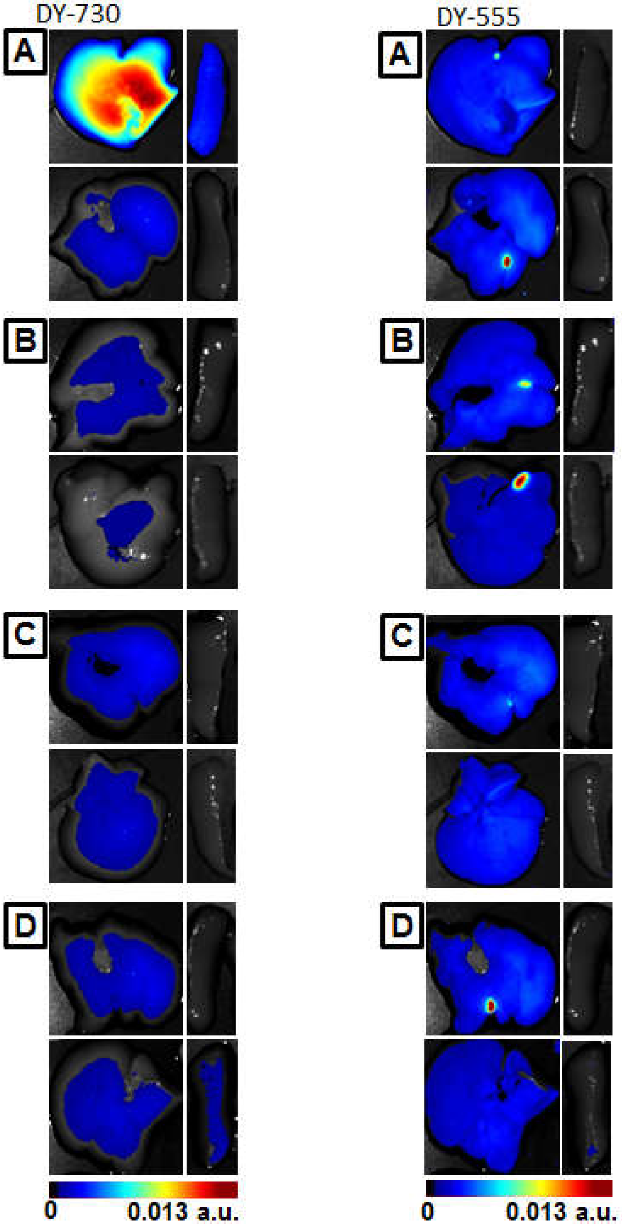

Figure S1. Macroscopic light and super-imposed color-coded fluorescence images of liver and spleen after injection of dually-labelled MNP-D(DY-730)-PEG-5kDa(DY-555). Left panel: Fluorescence of DY-730, right panel: fluorescence of DY-555. Nude mice received 200 μmol Fe/kg body weight.
